# The contribution of traditional healers' clinics to public health care system in Addis Ababa, Ethiopia: a cross-sectional study

**DOI:** 10.1186/1746-4269-7-39

**Published:** 2011-12-02

**Authors:** Wubet Birhan, Mirutse Giday, Tilahun Teklehaymanot

**Affiliations:** 1 College of Medicine and Health Sciences, University of Gondar P. O. BOX 196, Gondar, Ethiopia; 2 Aklilu Lemma Institute of Pathobiology, College of Health Sciences, Addis Ababa University, P. O. Box 1176, Addis Ababa Ethiopia

**Keywords:** medicinal plant, traditional healers, public health, Addis Ababa, Ethiopia

## Abstract

**Background:**

Ethiopian people have been using traditional medicine since time immemorial with 80% of its population dependent on traditional medicines. However, the documentation of traditional healers' clinics contribution to modern public health system in cosmopolitan cities is scanty. Studies conducted so far are limited and focused on the perceptions and practices of modern and traditional health practitioners about traditional medicine. Thus, a cross sectional study was conducted from February to May 2010 to assess the contribution of traditional healers' clinics to public health care system in Addis Ababa.

**Materials and methods:**

Ten traditional healers who were willing to participate in the study and 306 patients who were visiting these traditional healers' clinics were interviewed using two types of semi-structured questionnaires. Data were summarized using percentages, tables and bar chart.

**Results:**

The diseases mostly treated by traditional healers were wound, inflammation, herpes zoster, hemorrhoids, fracture, paralysis, back-pain, liver diseases, cancer and eczema. This study showed that traditional healers' clinics considerably contribute to public health care in Addis Ababa. Fifty two percent of patients reported that traditional healers' clinics were their first choice when they faced health problems. The reasons for visiting these clinics were 175 (57.2%) efficacy, 109 (35.6%) dissatisfaction with modern medicine, 10 (3.3%) dissatisfaction with modern medicine and efficacy, 6 (2.0%) cost and 6 (2.0%) dissatisfaction and cost. Females (55.2%), young age (20-40 years, 65.0%), never married (56.9%), orthodox (73.9%), Amhara (52.3%), educational status above grade 12 (34.6%) and government employees (29.4%) were frequent visitors. Healers reported that there was no form of cooperation with modern health professionals. The reasons were lack of motivation to collaborate and communicate with modern health service workers. Family based apprenticeship was the sources of knowledge for majority of the healers.

**Conclusions:**

The study conducted showed that for the majority of patients interviewed traditional healers' clinics were one of the options to solve their health problems that indicated the considerable contribution of these clinics to the public health care system in Addis Ababa. Nevertheless, in this study the contribution of traditional healers' clinics to the public health system would have been better shown if individuals who are not users of the traditional healers' clinics were included in the interview. However, the study might be useful as a base line data for future evaluation of the significance of traditional healers' clinics for public health system and the services rendered in these clinics.

## Background

Traditional medicine refers to health practices, approaches, knowledge and beliefs incorporating plant, animal and mineral-based medicines, spiritual therapies, manual techniques and exercises, applied singularly or in combination to treat, diagnose and prevent illnesses or maintain well-being [1]. Traditional medicine is commonly used to treat or prevent diseases including chronic illness therefore improving the quality of life. It occupies an important place in the health care systems of developing countries. It is estimated that more than 80% of health care needs in these countries are met through traditional health care practices [2,3].

A traditional healer is defined as an educated or layperson who claims ability or a healing power to cure ailments. He could have a particular skill to treat specific types of complaints or afflictions and might have gained a reputation in her/his own community or elsewhere. Traditional healer may base his power or practice on religion, the supernatural, experience, apprenticeship or family heritage [3-6].

In the last decade, there has been a global increase in the use of traditional and complementary/alternative medicines in both developed and developing countries [5]. The reasons in developing countries are cultural acceptability, perceived efficacy, affordability, accessibility and psychological comfort. The other factors are inaccessibility of modern health services in terms of geography, cost or time, shortage of well-trained modern health professionals [3,6-10].

Eighty percent of human and 90% of livestock in Ethiopia depend on traditional medicine for primary health care services where modern public health services are limited or note available [3,8-10]. Traditional healers play an essential role in the delivery of primary health care to local people as they treat people in resource poor settings. These people have poor access to modern health services and could not afford the cost for modern health services [3,6,9-11]. However, the contribution of traditional clinics to public health care system in Addis Ababa and other cosmopolitan cities where modern health services are found aggregated is not well documented and studies conducted so far are limited on the perceptions and practices of modern and traditional health practitioners about traditional medicine [6,12-16]. Therefore, the purpose of this study is to document the type of diseases treated by traditional healers, reasons for choosing traditional healers' clinics and magnitude of contribution of traditional healers to public health care system in Addis Ababa. The study might be useful as base line data for future evaluation of the significance of traditional healers' clinics for public health system and the services rendered in these clinics.

## Materials and methods

### Description of the study area

Addis Ababa is the capital city of Ethiopia with a population of 2.74 million [17]. Its area is estimated to be 530 Km^2 ^with altitudes ranging from 2200 to 3000 m above sea level, average temperature of 22.8°C and average rainfall of 1,180.4 mm. Addis Ababa has 30 hospitals, 29 health centers, 122 health stations, 37 health posts and 382 modern private clinics [18].

### Study subjects

Study subjects were 10 traditional healers who were willing to participate in the study, patients, 306, who were willing and attending traditional healers' clinics during data collection period in Addis Ababa.

### Ethnobotanical data collections

The ethnobotanical data were collected using two types of semi-structured questionnaires from February to May 2010: one for traditional healers' clinics clients and the other for traditional healers. Face to face, interviews were conducted with traditional healers and their clients, and individuals accompanying children less than five years. Information on demographic characteristics, use and types of traditional medicine, sources of healing knowledge, number of visitors per day, reasons for visiting traditional healers' clinics, and the common types of diseases treated by healers was collected. The semi-structured questionnaires were prepared in English and discussion with respondents was conducted in the local language, Amharic.

### Data analysis

Data were summarized using percentages and bar chart. Pearson's Chi-squares test was used to show presence or absence of association among different socio-demographic variables with traditional medicine use. P-value of less than 0.05 was considered as statistically significant difference. Single sample *t *test was conducted to determine variability within each category. SPSS version 13.0-computer software was used to analyze the data.

### Ethical clearance

The study was ethically approved by Institute Review Board of Aklilu Lemma Institute of Pathobiology, College of Health Sciences, Addis Ababa University. Prior to the initiation of the interview, the aim of the study was elaborated to the participants, verbal consents were obtained from both traditional healers, and their clients' that participated in the study.

## Results

### Socio-demographic characteristics of patients visiting traditional healers' clinics

Three hundred and six patients: 44.8% (137) male and 55.2% (169) female with a mean age of 28.1 years were interviewed and there was no significant difference between sexes (p > 0.05). The participants, young (20-40 years, 65.0%), never married (56.9%), orthodox (73.9%) and Amhara (52.3%) were frequent visitors of healers' clinics (single sample *t *test, p < 0.05) (Table 1). There was significant difference within each demographic category (Chi square test, p < 0.05).

**Table 1:** Socio-demographic variables of patients visited traditional healers' clinics in Addis Ababa, 2010

Variables	Number (%)	One-Sample Test			95% Confidence Interval of the Difference	
		**t**	**d.f**.	**Sig. (2-tailed)**	**Lower**	**Upper**

**Age in years**		1.699	5	0.150	-26.175	128.175
0-4	16(5.2)					
5 - 9	18(5.9)					
10 - 19	26(8.5)					
20-40	199(65.0)					
41-65	42(13.7)					
> 65	5(1.6)					
**Marital status**		1.993	2	0.184	-118.193	322.193
Never married	174(56.9)					
Married	129(42.2)					
Divorced	3(1.0)					
**Religion**		1.638	2	0.243	-165.919	369.919
Orthodox	226(73.9)					
Muslim	50(16.3)					
Protestant	30(9.8)					
**Ethnicity**		1.926	7	0.095	-8.709	85.209
Oromo	87(28.4)					
Amhara	160(52.3)					
Tigray	12(3.9)					
Wolayita	8(2.6)					
Guragie	13(4.2)					
Silte	12(3.9)					
Hdya	6(2.0)					
Others	8(2.6)					
**Educational status**		3.660	4	0.022	14.828	107.972
Illiterate	30(9.8)					
Adult education	18(5.9)					
1-6 grade	64(20.9)					
7-12 grade	89(29.1)					
> 12 grade	106(34.6)					
**Occupation**		5.023	7	0.002	20.243	56.257
Business	38(12.4)					
Daily laborer	31(10.1)					
Farmer	11(3.6)					
Government employee	78(25.5)					
House wife	31(10.1)					
Jobless	16(5.2)					
Private institution	47(15.4)					
Student	54(17.6)					
**House hold income**		8.386	3	0.004	41.574	92.426
< 500	65(21.2)					
500-850	59(19.3)					
851-1500	90(29.4)					
> 1500	54(17.6)					

### Health seeking behavior of patients visiting traditional healers' clinics

**Figure 1:**
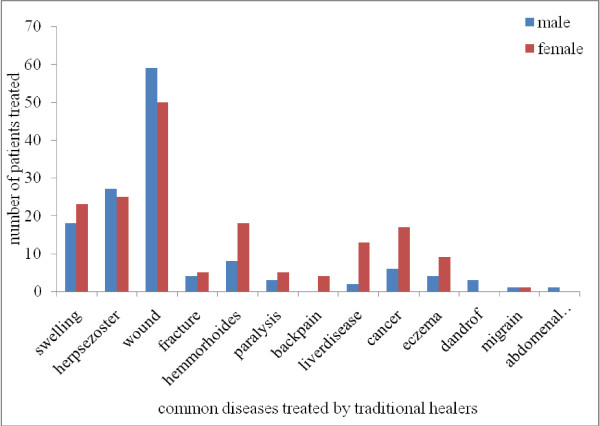
**Diseases frequently treated by traditional healers in Addis Ababa, 2010**.

Traditional healers' clinics were first choice for 172(56.2%) patients for diseases like swelling, herpes zoster, wound, fracture, hemorrhoids, paralysis, back-pain, liver diseases, cancer and eczema (Figure 1). One hundred nineteen (38.9%) patients got information about traditional healers clinics from friends, 80(26.1%) from family, 61(19.9%) from previously treated individuals and 46(15.0%) form multiple sources. Most patients, 183(59.8%) visited traditional healers' clinics escorted by their family, whereas 96(31.4%) went by themselves, and 27(8.8%) with friends. One hundred seventy and three (56.5%) patients visited healers' clinics once, 85(27.8%) two times, 34(11.1%) three times, 9(2.9%) four times and 5(1.6%) more than 4 times in their life time.

### Reasons of visiting traditional healers' clinics

Seventy-four (24.2%) patients that visited traditional healers' clinics reported that they were previously treated and cured. They were treated of diseases such as herpes zoster 35(11.4%), wound 22(7.2%), eczema 11(3.6%) and swelling six (2.0%). The reasons for visiting traditional healers' clinics by patients were 175(57.2%) efficacy, 109(35.6%) dissatisfaction with modern medicine, 10(3.3%) dissatisfaction with modern medicine and efficacy, 6(2.0%) cost and 6(2.0%) dissatisfaction and cost.

### Attitude of patients to traditional healers' clinics

The majority (65.4%) of the patients had positive attitude to the efficacy of traditional medicine and out of these patients, 116 (37.9%) rated the efficacy of traditional healers' service as good and 177 (57.8%) indicated side effect was low. About Fifty-nine percent of patients reported that they were satisfied with traditional healers' clinics services (single sample *t *test, p < 0.05) (Table 2).

**Table 2:** Attitude of patients visited traditional healers' clinics towards traditional healers' clinics in Addis Ababa, 2010

Variables	Number (%)	One-Sample Test			95% Confidence Interval of the Difference	
		**t**	**d.f**.	**Sig. (2-tailed)**	**Lower**	**Upper**

**Efficacy of traditional medicine(TM)**		4.31	3.00	0.02	20.02	132.98
Very good	84(27.5)					
Good	116(37.9)					
Fair	30(9.8)					
Difficult to decided	76(24.8)					
**Side effect of TM**		1.30	2.00	0.32	-161.72	301.06
No	177(57.8)					
Dose	22(7.2)					
It is not known	107(35.0)					
**Advise to visit TM**		2.77	2.00	0.11	-56.58	260.58
Yes	172(56.2)					
No	47(15.4)					
Difficult to decide	87(28.4)					
**Satisfaction**		2.48	2.00	0.13	-75.21	279.21
Yes	182(59.5)					
No	45(14.7)					
Difficult to decide	79(25.8)					

### Socio demographic characteristics of traditional healers

The interviewed healers were males with a mean age of 51 years, and had religious education. They started traditional healing practice at their young age (20-40 years) and were generalists that were treating different types of diseases. The majority (70%) of healers were Orthodox Christians. Their sources of knowledge (50%) were apprenticeships to parents (Table 3).

**Table 3:** Socio demographic data on traditional healers in Addis Ababa, 2010

Socio demographic data	Number (%)
**Age in years**	
20-40	3(30.0)
41-65	6(40.0)
> 65	1(10.0)
**Marital status**	
Single	5(50)
Married	5(50)
**Educational status**	
Religious education	4(40.0)
Religious education and grade 1-6	3(30.0)
Religious education and grade 7-12	3(30.0)
**Types of practice conducted**	
Herbalist	3(30.0)
Herbalist and bonesetter	7(70.0)
**Source of traditional healing practice**	
Apprentice hood to parents	5(50.0)
Family, apprentice hood with other person	4(40.0)
God gift, apprentice hood with other person	1(10.0)
**Job other than TM**	
Yes	6(60.0)
No	4(40.0)
**Do you had work before TM**	
Yes	7(70.0)
No	3(30.0)

### Knowledge and Practice of healers in Addis Ababa

The majority (70.0%) of healers identified diseases and causes of illness by history-taking and physical diagnosis. During history taking, patient or person accompanying the patient was interviewed about the sign and symptoms of disease, the duration of the disease, age of the patient and history of similar disease in the family. In examination, they observed signs of diseases such as face color, abdomen size and discomfort, wound size and site, and urine color. On the other hand, a minority (30.0%) of the healers were using combination of history-taking, physical diagnosis and divination in identifying diseases and determining the type of medication.

### Source of medicine, preparation, prescription and fee

The sources of medicine for the majority of interviewed traditional healers were plants, animals and minerals while for two healers were plants and animals, and for one healer were only plants. Two of the healers had home-gardens for cultivation and as source for some medicinal plants. All healers used both dry and fresh plants parts for preparation of remedies. Crushing, powdering and pounding were indicated by six of the healers as the methods of preparations of herbal drugs, while four of the healers only used squeezing. All healers stored medicinal plants in the form of powder or dried and cut into pieces within a closed container. The time of storage varied among the healers and depended on the type of traditional medicine. The doses of the medicine were measured using cup, spoon, glass, pinch, and lid of the container; it was determined by age of the patient, physical status of the patient, severity of the disease and the experience of individual healer.

All healers had offices for their healing practice but none of them admitted and treated inpatients. Seven (70%) healers responded that they had additional persons working with them as assistant healers, their number ranged from 1 to 5. Healers received payment for their services that included registration fee and cost of medicine. The registration fee ranged from 2.00 birr to 20.00 birr (1 ETB = 0.04 EURO, 0.06 USD) though none of the traditional healers' have formal registration system for their patients. The cost of medicine was paid immediately after getting the treatment and showed variation from healer to healer as well on type of disease

### Referral, collaboration and feed back

One of the healers responded that he had referred patients to modern health institution and to 'spiritual wholly water' treatment when the illness of the patient was beyond his professional capacity and skill. All interviewed healers did not get help from modern health professionals and did not initiate cooperation with modern health professionals. The reasons mentioned were lack of motivation to collaborate and communicate with modern health service workers and vice versa. The majority (60.0%) of the healers got feedback from their customers on areas such as the strength of their service, efficacy, fees of treatment and medicine.

## Discussion

The number of individuals found in the traditional healers' clinics during data collection period and who responded those traditional healers' clinics as their first choices could indicate the contribution of traditional healers' clinics to the public health system. The number of repeated visits of these clinics by patients and number of individuals that gave information to the patients about traditional healers' clinics that might have previously visited traditional healers' clinics also demonstrated the significance of the traditional healers' clinics for the public health system in Addis Ababa. These showed that a considerable number of the population was treated by the traditional healers' clinics and hence, the contribution of these clinics to public health systems in Addis Ababa.

The majority of patients in this study preferred traditional health care clinics than modern health facilities. Females, individuals with middle-income level and those with education visited traditional healers' clinics more frequently than the rest of informants. This is in agreement with the study done in Trinidad [19]. However, it is different from the studies conducted in California [20], Israel [21] and Colombia University [22] where females, those with higher education and high-income level had statistically significant association with traditional medicine use. In most studies, low income has been mentioned as the reason to visit traditional healers' clinics [9-11] whereas in this study it was not found as a determinant in visiting traditional healers' clinics since other categories were equally important, which was indicated by single sample *t *test distribution (Table 2).

Reasons indicated by patients that participated in the present study for using traditional medicine as their first choice when they were ill is similar to the study done in Trinidad [19] where efficacy of traditional medicine was the reason for choosing herbal medicine as the first line of health care option. This high efficacy perception may be because traditional medicine was embedded in the belief and culture of the society [9-11]. On the other hand, the study conducted in Addis Ababa to determine the epidemiology of herbal drug use [13] showed that the main reasons given for choosing herbal medicine as the first line medication option were dissatisfaction with the services of modern health institutions due to their time-consuming practice, cost and perceived efficacy. Study conducted in Nigeria [23] also agrees with the present study that high efficacy of traditional medicine and dissatisfaction with modern medicine were the reasons to visit traditional healers' clinics.

The study conducted in the United States [24] to investigate possible predictors of alternative health care use indicated that those with higher education and poorer health status were associated with alternative medicine use. This is not in agreement with the current study, however level of education had a contribution in visiting traditional healers' clinics.

A majority of patients, in this study, visiting traditional healers' clinics were associated with dermatological cases. Study conducted in Pakistan [25] showed that 43% of the patients preferred traditional healers for skin disorder treatment indicating that the effectiveness of the remedies given by traditional healers against dermatological diseases.

The finding of this study that majority of patients were satisfied after being treated by traditional healers is corroborated by the study conducted in Zambia [26] and Tanzania [27]. The study conducted in Nigeria [23] indicated that 33.4% of the respondents reported that herbal medicines had no adverse effects though lower than the current study. The difference could be due to the variation in the dosage and the type of herbs used.

The source of the healers' knowledge in this study is similar to the study conducted in Tanzania [27] where for 41.9% of the healers were their families. On the other hand most healers in Tanzania kept patient records containing demographic, diagnosis and treatment data whereas in the current study none of the healers kept patient records. The healers in the current study followed traditional treatment systems. Healers in Tanzania [27] agree in diagnosis of patients with this study though they also use laboratory test results made in the hospital in addition to history taking, physical diagnosis, and divination to identify diseases.

In the current study, only one healer referred his patients to modern medicine but the study done in Tanzania (27) showed that almost all healers referred their patients to hospitals when they failed with their own treatment. This difference may be because absence of collaboration and lack of training of traditional healers in Addis Ababa.

## Conclusion

The study conducted showed that for the majority of patients interviewed traditional healers' clinics were one of the options to solve their health problems, which indicated the considerable contribution of these clinics to the public health care system in Addis Ababa. The main reasons for choosing traditional healers' clinics were efficacy, safety of the traditional medicines and affordability of the services provided by the healers' clinics. Nevertheless, in this study the contribution of traditional healers' clinics to the public health system would have been better shown if individuals who are not customers of the healers' clinics were included in the interview. However, the study might be useful as a base line data for future evaluation of the significance of traditional healers' clinics for public health system and the services rendered in the healers' clinics.

## Competing interests

The authors declare that they have no competing interests.

## Authors' contributions

The authors have made substantive intellectual contributions to this study in data collection, identification of plants, preparation of the manuscript and all authors have read and approved the final manuscript.
